# Higher-molecular-weight a-synuclein oligomers are increased in the brain cytosol of patients with dementia with Lewy bodies

**DOI:** 10.1038/s41531-026-01301-2

**Published:** 2026-02-28

**Authors:** Emil Gregersen, Mia R. Antorini, Lasse Reimer, Ludovica Zaccagnini, Dennis Selkoe, Tim Bartels, Poul Henning Jensen

**Affiliations:** 1 Danish Research Institute of Translational Neuroscience – DANDRITE, Aarhus University, Aarhus, Denmark; 2 Department of Biomedicine, Aarhus University, Aarhus, Denmark; 3 Department of Neurology, UK Dementia Research Institute, University College London, London, UK; 4 Department of Neurology, Ann Romney Center for Neurologic Diseases, Brigham and Women’s Hospital, Harvard Medical School, Boston, MA USA

**Keywords:** Biochemistry, Neurology, Neuroscience

## Abstract

The demonstration of MJFR14-6-4-2 proximity ligation assay positive aggregates in brains affected by Parkinson’s disease and LRRK2 mutations, motivated the analysis of α-synuclein oligomers in brain cytosol from dementia with Lewy bodies (DLB) and control cases by combining size-exclusion chromatography, aggregate-specific MJFR14-6-4-2 ELISA and SDS-denaturing immunoblotting. We demonstrate a DLB-specific accumulation of large SDS-soluble oligomers, and the presence of medium-sized oligomers consisting of SDS-resistant α-synuclein dimers and trimers in both DLB and controls.

Aggregation of the presynaptic protein α-synuclein (a-syn) is tightly associated with Parkinson’s disease (PD) and dementia with Lewy Bodies (DLB), based on genetic and biochemical evidence^1–3^. The aggregates are present as insoluble amyloid-type fibrillar species hyperphosphorylated on Ser129, typically associated with Lewy body (LB)-type inclusions, and soluble oligomers^4,5^. High-resolution cryo-EM tomography studies have revealed detailed structural insight into disease-associated fibrillar strains^6^. However, our insight into the nature of the oligomers in the brain is lacking in comparison, despite oligomers having been hypothesised to be pathogenic for decades^7–9^, and in vitro-generated oligomers have been studied in detail^10,11^. The existence of oligomers and their increase in extracts of human brains affected by synucleinopathies have been demonstrated by different biochemical assays^12–14^, and in the cytosol from DLB brains by enzyme-linked immunosorbent assay (ELISA^15^).

Recent immunochemical studies take advantage of a-syn proximity ligation assays (PLA) to detect a-syn oligomers. Two protocols have been developed, both using pairs of either the monoclonal pan-a-syn antibody Syn211 or the aggregate-specific MJFR14-6-4-2 antibody^16–20^. Using the MJFR14-6-4-2 PLA, it was demonstrated that oligomers are abundant in tissue from PD brains, where they develop prior to the formation of LBs, but are absent or in low abundance in non-synucleinopathy control brain tissue. Moreover, they exist in brains without LBs from patients with mutations in the LRRK2 gene^18,20^.

The size distribution of total a-syn species in the cytosol of DLB and control brains has been analysed by size-exclusion chromatography (SEC), followed by total a-syn ELISA of the individual cytosolic fractions^21^. This demonstrated a symmetric peak with a maximum corresponding to a molecular size of 150 kDa and negligible levels larger than 300 kDa. The fractions from the gel filtration study covered the molecular size range from >2000 kDa to approximately 30 kDa and were categorised into three groups: A low molecular weight (LMW) pool in fractions 11–13 of ~50–200 kDa, an intermediate-molecular-weight (IMW) pool in fractions 8–10 of ~ 250–400 kDa, and a high molecular weight (HMW) pool in fractions 2–7 > 450 kDa (Figs. 1–3).

**Fig. 1:**
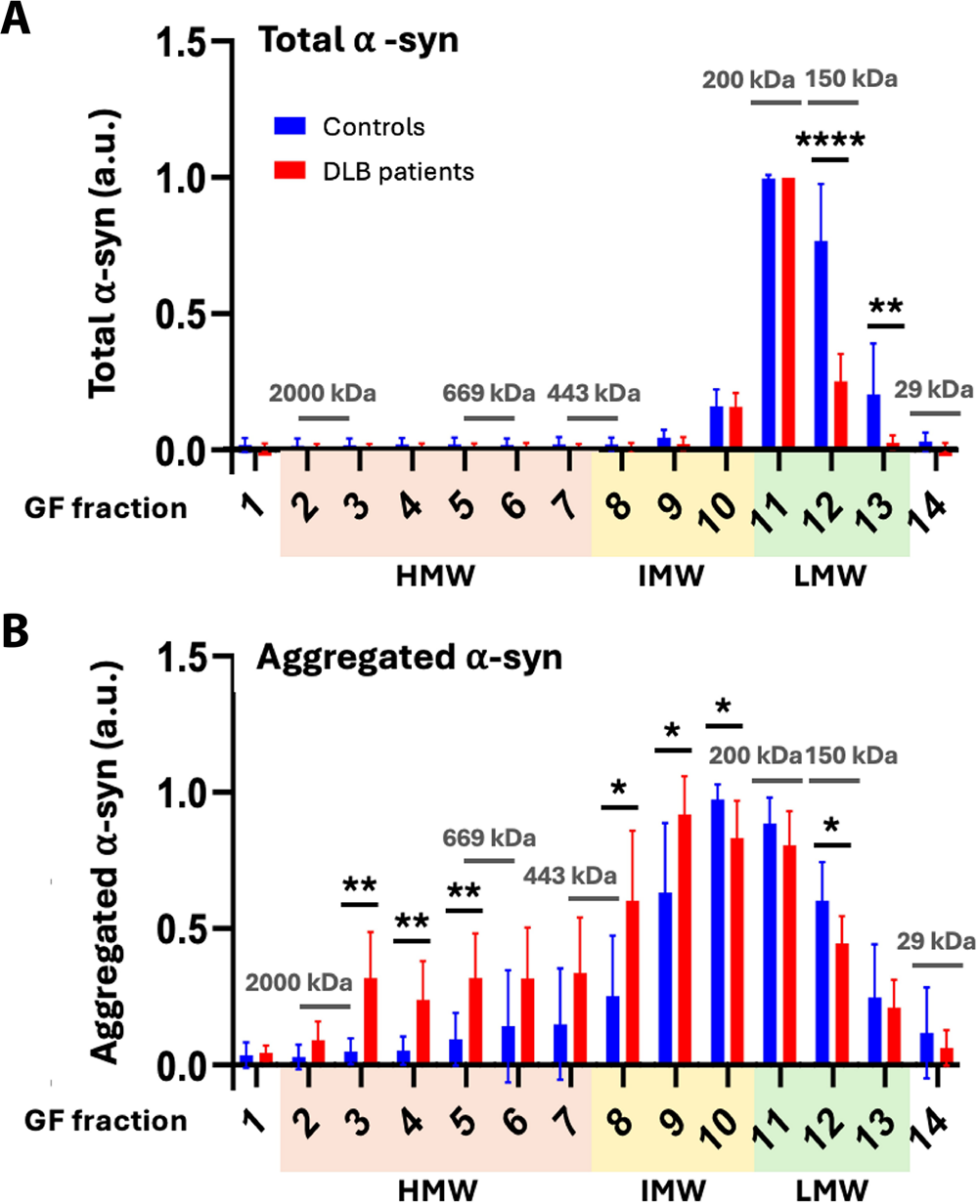
Detection of increased oligomerization in DLB cortex by α-syn aggregate-specific ELISA. Brain homogenates from dementia with Lewy body (DLB) patients or neurologically healthy controls (CTRL) were separated by size-exclusion chromatography (SEC). The gel filtration fractions (GF) were divided into three pools based on their molecular size: a low molecular weight (LMW) fraction corresponding to ~50–200 kDa, an intermediate-molecular-weight (IMW) fraction of ~250–400 kDa, and a high-molecular-weight (HMW) fraction of >450 kDa. The fractions obtained were analysed by ELISA for the content of total a-syn (**A**), and aggregated a-syn (**B**) using the polyclonal ASY-1 and monoclonal a-syn aggregate-specific MJF14-6-4-2 rabbit IgG as primary antibodies, respectively. Each set of fractions from individual patients were diluted until the signal was in the linear part of the standard curve (usually 156 pg/mL–20 ng/mL). To compare SEC profiles, fraction measurements were normalised to the highest measurement within that individual set of fractions. Control samples are depicted in blue and DLB in red. The molecular size markers are based on the calibration presented in Sanderson^21^ where the samples were initially isolated. The mean values of CTRL and DLB are displayed with their standard deviations (*n* = 8). Statistical significance was determined by multiple unpaired t-test with Welch’s correction (*p < 0.05, **p < 0.01, ****p < 0.0001).

**Fig. 2:**
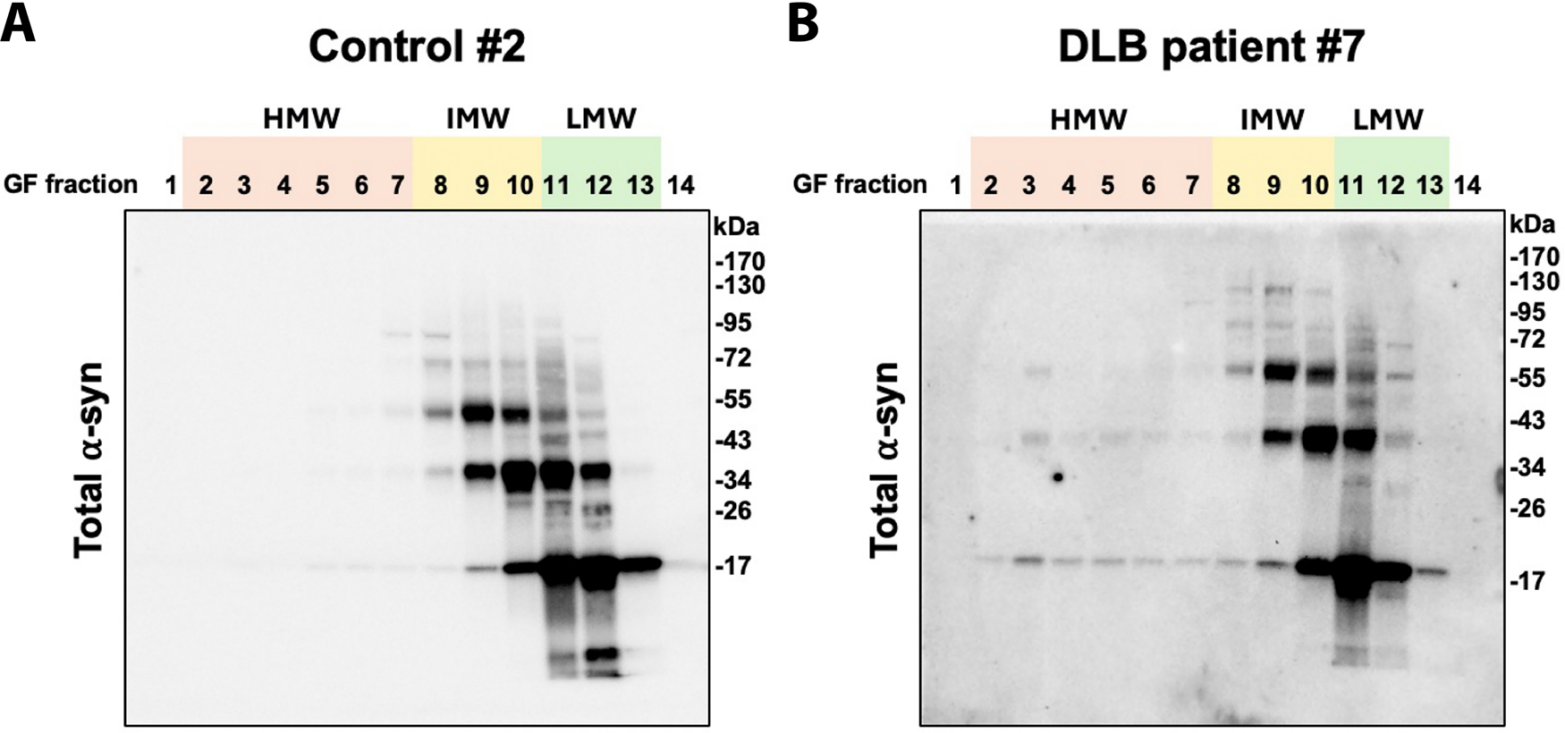
IMW oligomers are more SDS-stable than HMW oligomers. Brain homogenates from dementia with Lewy body (DLB) patients or healthy controls (CTRL) were separated by SEC. The collected fractions were resolved by denaturing SDS-PAGE gel, followed by western blot analysis using the polyclonal antibody ASY-1. A protein ladder was used as a molecular size marker and indicated to the right in kDa. The blots represent CTRL (#2) in (**A**) and DLB (#7) in (**B**) and are representative of their groups (see Supplementary Fig. 1a–d). The numbers of the GF fractions and their grouping into LMW, IMW and HMW pools are indicated on top of the images. Please note that the α-syn bands in HMW fractions 3–5 in the DLB case are almost exclusively monomeric and not detectable in the CTRL case. By contrast, the α-syn bands in fractions 8–10 are dominated by dimeric and trimeric species. This demonstrates that the HMW α-syn oligomer species in the DLB cases are sensitive to denaturation by SDS, whereas the smaller oligomers in fractions 8–10 predominantly dissociate into trimers and dimers.

**Fig. 3:**
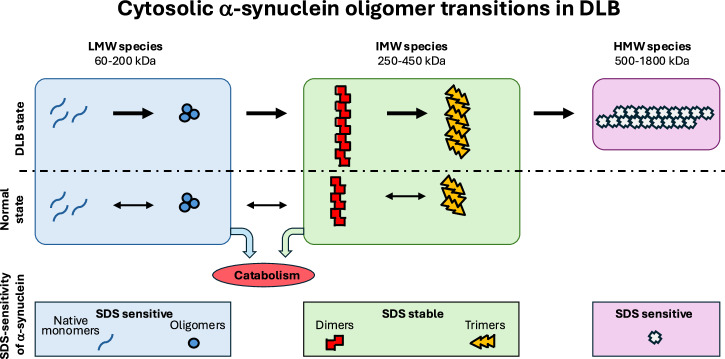
Hypothesis: Cytosolic oligomer transitions in DLB. We hypothesise that native cytosolic a-syn under normal homoeostatic conditions exists in equilibrium with oligomeric pools that are detectable by MJFR14-6-4-2 aggregate ELISA. The LMW pool elutes with the majority of total a-syn and dissociates into an SDS-sensitive 15 kDa species in SDS-PAGE (light blue box). The dominant IMW pool elutes earlier than the fractions containing the majority of total a-syn. These species dissociate into SDS-resistant dimers and trimers by SDS-PAGE indicative of posttranslational modifications (PTM), potentially oxidative crosslinks (green box). The LMW and IMW species exist in the normal state and are likely substrates for normal catabolic mechanisms (red circle) and the PTM causing the SDS-resistant dimers and trimers could represent signals for degradation. In DLB, the IMW oligomers increase further in size due to elongation with native 15 kDa monomers and ultimately form large SDS-sensitive HMW oligomers of more than 1000 kD. The DLB associated build-up of large IMW species and HMW species will lead to progressive cellular dysfunctions. This build-up can be caused by either compromised catabolic systems, e.g. (i) lysomal function, (ii) a decreased cellular ability to form the SDS-resistance promoting PTM’s that promote oligomer degradation or, (iii) a combination hereof. The MJFR14-6-4-2 PLA-positive aggregates detected in brain tissue from patients affected by sporadic PD and LRRK2 mutant PD were more abundant than in the control cases, although typically weaker signals indeed were present in some control^17,18^. Based on our GF analysis and aggregate ELISA, the larger IMW and all the HMW oligomers are candidates for the PLA-positive structures detected in tissue.

In this study, we hypothesised that insight into the size of MJFR14-6-4-2 positive a-syn aggregates in DLB brain cytosol would be informative as to the size of the candidates responsible for the MJFR14-6-4-2 PLA signal in DLB brain tissue (Fig. 1). Moreover, reducing SDS-PAGE analysis and anti-a-syn immunoblotting will have the power to demonstrate if major differences exist in the resistance of a-syn aggregates to denaturation between the a-syn oligomer pools (Fig. 2). The 8 neurologically healthy controls and 8 DLB patients to be studied (Table 1) were chosen from a larger cohort previously analysed for the presence of total a-syn^21^.

**Table 1:** Demographics and clinical characteristics of the DLB cohort and controls included in this study

	Case #	Source	Diagnosis	Age	Sex
DLB 1	20070105	Newcastle	DLB	71	M
DLB 2	Syn2	Mayo Clinic	DLB	67	F
DLB 3	Syn3	Mayo Clinic	DLB	68	F
DLB 4	1594	MGH	DLB	81	F
DLB 5	Syn6	Mayo Clinic	DLB	54	M
DLB 6	A01-213	Brigham and Women’s	DLB	83	M
DLB 7	1650	MGH	DLB	76	M
DLB 8	1751	MGH	DLB	87	F
CTRL 1	P48/07	Queen Square	Cog. normal control		F
CTRL 2	CON 2	Mayo Clinic	PA	75	F
CTRL 3	CON 3	Mayo Clinic	PA	81	F
CTRL 4	1901	MGH	Control	54	M
CTRL 5	1887	MGH	Control	60	M
CTRL 6	1821	MGH	Control	92	M
CTRL 7	CON 7	Mayo Clinic	SC/VaD	79	F
CTRL 8	CON 8	Mayo Clinic	SC	70	M

Grey matter from the frontal cortex of DLB patients and corresponding controls was analysed.

*F* female, *M* male, *MGH* Massachusetts General Hospital, *NA* not applicable, *PA* pathological aging, *PMI* post-mortem interval, *SC* senile changes, *VaD* vascular dementia.

Quantifying the samples isolated from DLB and control brains (Table 1), by SEC and MJFR14-6-4-2 ELISA^22^ demonstrated that both control and DLB brains contain significant amounts of LMW and IMW a-syn species (~150–400 kDa), which were shifted towards HMW fractions in DLB patients (Fig. 2B). The larger HMW oligomers in the range of ~500–1800 kDa appeared selectively increased in DLB. This suggests that large cytosolic oligomers are candidates for the structures generating the MJFR14-6-4-2 PLA-positive signals in synucleinopathies, although other species present in other brain fractions, e.g., bound to vesicles or organelles, may also contribute.

Our total a-syn ELISA demonstrated similar elution profiles for both control and DLB cytosol, with the vast majority of a-syn eluting as a symmetric peak with a maximum in the LMW fraction with maximum corresponding to a molecular size of ~200 kDa (Fig. 1A). There is no substantial signal in the IMW and HMW fractions below fraction 10. This molecular size profile is in agreement with the previous measurement of the larger cohort, from which the 8 controls and 8 DLB patients were chosen^21^.

Compared to the total a-syn ELISA, the aggregate-specific MJF14-6-4-2 ELISA detected a broader peak, with oligomeric a-syn spanning the entire IMW and LMW fractions in control patients (Fig. 1B). This LMW fraction was shifted to IMW sizes in the DLB cytosol, with a maximum in fraction 9 (~340 kDa) compared to fraction 10 (~250 kDa) for the controls. In addition to the IMW fraction, a pool in fractions 2–7 could be found in the DLB samples as a prominent HMW “shoulder” on the IMW peak. This contrasted with the controls, where this HMW “shoulder” was smaller and tapered off in fractions 5–7. Hence, there exists a quantitative difference between the HMW pool of a-syn oligomers in DLB and controls, where it accounts for 25.7% ± 4.9 compared to 10.2% ± 4.8 of the total aggregated a-syn signal (calculated based on area under the curve). This may indicate increased aggregation or reduced catabolism of oligomers in the DLB patient’s frontal cortical grey matter, resulting in cytosolic accumulation of larger oligomers with molecular sizes of ~500–1800 kDa. This oligomer pool is low in abundance compared to the total amount of a-syn as the total amount of a-syn in the HMW fractions of DLB cytosol is not significantly higher than in control (Fig. 1A), which agrees with the analysis of the larger cohort wherefrom our samples were drawn^21^. Despite the low amount of a-syn in the HMW fraction of DLB patients, they exhibited a stronger vesicle permeating activity than from controls, which could be neutralised with an antibody toward a-syn^21^. This suggests that even low amounts of soluble cytosolic aggregated a-syn possess novel potent functions as previously demonstrated by strong inhibition of the 20S proteasome^23^. A deeper analysis of such effects will require purification of larger amounts of these cytosolic fractions but this should be possible because the cytosolic fractions represent ~80% of total a-syn in brain tissue with less than 20% being membrane associated^21^. As an orthogonal analysis to the quantitative ELISA, we subjected the SEC fractions to reducing SDS-PAGE and immunoblotting with a polyclonal a-syn antibody, ASY-1^24,25^. Figure 2A, B demonstrates representative immunoblots of cytosol fractions from patients, Control #2 and DLB #7. The immunoblots from the remaining patients are presented in Supplementary Fig. 1a–d. The LMW pool is dominated by SDS-sensitive species dissociating into 17 kDa monomers upon denaturation in the loading buffer for the SDS-PAGE. The IMW pool is dominated by species dissociating into SDS-resistant dimers and trimers. The LMW and IMW fractions do not differ noticeably in their ASY-1 immunoreactive patterns between controls and DLB patients (Fig. 1A), although it is noteworthy that the IMW pool represents the fractions wherein the ELISA-positive oligomers accumulated in DLB patients compared to controls (Fig. 2B).

The HMW pool is weakly positive on the immunoblots of both DLB and controls but is evidently occupied by 17 kDa monomeric a-syn species, thus indicating higher sensitivity to denaturation by SDS compared to the IMW pool (Fig. 2B). To better appreciate this pool, we present longer exposures of the immunoblots (Supplementary Fig. 1a–d).

The existence of a significant fraction of IMW-sized a-syn oligomers in cytosol from frontal cortex being composed of SDS-resistant dimers and trimers raises several questions. (1) What is the molecular basis for the SDS-resistance of these small polymers? In vitro studies have demonstrated oxidative crosslinks between C-terminal tyrosine’s predominantly yields low number polymers like dimers^26^. Careful proteomic studies of a-syn purified from brain cytosol by SEC could provide a strategy for identifying whether such crosslinks or other modifications form the basis for the dimers and trimers observed in Fig. 2A, B. The presence of dimers and trimers in controls suggests they could represent species that naturally are being catabolized and the crosslinking could represent a signal for their turn-over. By contrast, the aggregates in the HMW fractions predominantly dissociate into monomers. This could suggest they are generated by a different aggregation pathway that is unable to tag the monomers with signals that can facilitate their degradation.

Based on our data we hypothesise the existence of a pathway for a-syn aggregation in the cytosol of brain tissue that comprise a normal pool of MJFR14-6-4-2-positive oligomers, present under homoeostatic conditions in controls, which can grow into larger DLB specific oligomers (Fig. 3). The existence of SDS-resistant dimers and trimers in the homoeostatic pool suggests oligomer specific PTM may be involved in the catabolism of oligomers under homoeostatic conditions.

A strength of our study is the combination of (i) SEC, (ii) an a-syn aggregate-specific ELISA and (iii) reducing SDS-PAGE and a-syn immunoblotting on a subfraction of a well-characterised cohort of control and DLB brains. Our data demonstrate that a-syn aggregates, which bind the aggregate-specific MJFR14-6-4-2 antibody, are present in both control and DLB brain cytosol, but their average size increases in DLB. Meanwhile, the very large cytosolic oligomers 500–1800 kDa are exclusively present in DLB brains and are thus candidates for generating the MJFR14-6-4-2 PLA signal recently demonstrated to develop in brains affected by synucleinopathies (Fig. 3). The IMW-sized oligomers are dominated by a-syn building blocks consisting of SDS-resistant dimers and trimers caused by yet unknown PTM. These observations outline an experimental strategy to enrich soluble a-syn aggregates from cytosol and other brain fractions to provide the basis for molecular characterisation of these PTM and their potential role in oligomer catabolism, and ultimately structural insight into the oligomers e.g. by cryoEM tomography. A key question is to determine if such dimer- and trimer-rich species exist in cytosol fractions of cellular and animal models as this will facilitate such studies. SDS-sensitive species compatible with dimers and trimers were detectable in the endoplasmic reticulum/microsome fraction of sick but not healthy mPrp-h-A53T-transgenic mice, suggesting they also may occur in cytosol^27^.

A limitation of our study is its exploratory nature, as it only studied a small cohort and focused on the cytosolic fraction. Our findings motivate further studies using similar approaches across all subcellular fractions of brain tissue to provide greater insight into the anatomical, subcellular and size distribution of oligomers. For example, a previous study demonstrated that aggregated a-syn species were associated with ER membranes in PD^27^. One goal would be to develop protocols for oligomer purification that enable high-resolution cryo-EM tomography to solve the structures of these hitherto elusive pathogenic a-syn aggregates.

## Methods

### Brain samples and size exclusion chromatography

Human brain tissue from *post-mortem* brains of 8 healthy controls and 8 patients with a neuropathological diagnosis of DLB was sampled from the cohort previously analyzed^21^. Human brain tissue was provided by Brigham and Women’s Hospital (Boston, MA, USA), Mayo Clinic (Jacksonville, FL, USA), Massachusetts General Hospital/Massachusetts Alzheimer’s Disease Research Center (Boston, MA, USA), Newcastle Brain Tissue Resource (Newcastle upon Tyne, UK) and Queen Square Brain Bank for Neurological Disorders (London, UK). Information about the brain samples used in this study is summarised in Table 1. Consent was obtained from patients prior to death at each brain collection centre. All five brain banks approved of the proposal for the use of human tissue in this study, and the IRB and REC at the first and last authors’ institution deemed the planned use of this tissue to be appropriate and ethical (IRB 1999P001180/BWH, REC 18/LO/0721).

Approximately 500 mg of grey matter frontal cortex tissue pieces was homogenised with 25 strokes using a manual Dounce homogeniser overhead stirrer (Wheaton, Millville, NJ, USA) in four volumes (weight: volume) Tris-buffered saline (TBS)/protease inhibitor (PI) (20 mM Tris–HCl, 500 mM NaCl, pH 7.5 with complete PI tablet; Sigma- Aldrich, St. Louis, MO, USA). Homogenates were centrifuged for 5 min at 1000 × *g* at 4 °C to remove highly insoluble structures and debris from the tissue. The supernatant was subjected to an additional centrifugation at 175.000 × *g* for 30 min. 1–2.5 mg total protein from the resulting supernatant was subjected to SEC on a Superose 6 Increase 10/300GL size-exclusion column (GE Healthcare) using an ÄKTA chromatography system (GE Healthcare). The separation was carried out in 50 mM ammonium acetate, pH 7.4, at a flow rate of 1.5 mL/min. Each collected fraction of 1 mL was frozen in liquid nitrogen and stored at −80 °C. Molecular size was estimated based on a gel filtration molecular marker kit ranging from 29 to 700 kDa (Sigma-Aldrich, MGGF1000).

### Aggregated and total a-syn ELISA

ELISAs detecting aggregated and total a-syn were performed as previously described^22^. As a capture antibody, the a-syn aggregate ELISA utilised the aggregate-specific antibody MJF14-6-4-2 (Abcam, ab209538)^22,28^, while the total a-syn ELISA applied our in-house-made affinity-purified rabbit polyclonal ASY-1 antibody^24,25^ (0.26 µg/mL). Both ELISAs utilised Syn-1 (BD Transduction Laboratories, 610787) as the detection antibody. Fractions were diluted in 50 mM ammonium acetate, pH = 7.4, such that the strongest signal was above 5 ng/mL and did not exceed the linear part of the standard curve (usually between 156 pg/mL and 20 ng/mL). The standard curves were obtained using purified recombinant human monomeric and oligomeric a-syn as previously described^22^. To allow comparison of individual patient SEC profiles of total and aggregated a-syn, each fraction measured within a patient’s SEC profile was normalised to the fraction measurement with the highest level of analyte within this individual patient sample set. This allowed all profiles to be presented with values between 0 and 1, despite variations in their concentrations of total and aggregated a-syn.

### SDS-PAGE and western blot

10 μl fraction sample was combined with 2× SDS-loading buffer (20 mM Tris, pH 6.8, 2 mM EDTA, 2% SDS, 20% sucrose, 80 mM DTE) and boiled for 5 min at 96 °C. Samples were subjected to SDS-PAGE using Bis-tris 8-16% gels (Genscript, M81615) together with a molecular size marker (ThermoFisher, 26616). Next, proteins were transferred to a PVDF membrane using an IBlot™ 2. The membrane was fixed in 4% PFA for 30 min at RT, boiled for 5 min in PBS, and blocked in blocking buffer (5% skimmed milk powder, 20 mM Tris base, 150 mM NaCl, 0.05% Tween 20, 0.02% NaN_3_) for 1 h at RT. Incubation of polyclonal ASY-1 rabbit IgG^24,25^ as a primary antibody, detecting total a-syn was carried out in blocking buffer overnight at 4 °C. To validate the band pattern obtained with the ASY-1 IgG, the filter was also probed with a mouse monoclonal Syn-1 antibody (610787, BD Transduction Laboratories), which yielded similar results (data not shown). Incubation with secondary antibody conjugated to horseradish peroxidase was performed in blocking buffer without NaN_3_ for 1 h at RT. Thereafter, the membrane was washed 3× 5 min in tris-buffered saline (TBS, 0.05% Tween 20). Bound antibodies were visualised by ECL™ (GE Healthcare, RPN2209) and captured in a Fuji Las-3000 intelligent dark box (Fujifilm). Western blots were quantified using ImageJ2 (version. 2.14.0).

### Statistics

Fractions were analysed in pairs, assuming a Gaussian distribution without assuming a consistent standard deviation. Comparisons of multiple groups were analysed by multiple unpaired *t*-tests with Welch’s correction. Statistical significance was defined as a *p*-value < 0.05. All statistical analyses were performed using GraphPad Prism (GraphPad Software 9.2.0).

## Supplementary information

Supplementary information

## Data Availability

Data is provided within the manuscript, and raw data for the western blot analyses can be found in the supplementary information files.
