# Supplementary material for: Higher-molecular-weight a-synuclein oligomers are increased in the brain cytosol of patients with dementia with Lewy bodies
Source: NPJ Parkinsons Dis. 2026 Feb 28;12:89. doi: 10.1038/s41531-026-01301-2 (PMC13065805; doi:10.1038/s41531-026-01301-2)
Supplement: Supplementary file 1 — Supplementary information [file 41531_2026_1301_MOESM1_ESM.pdf]

a)

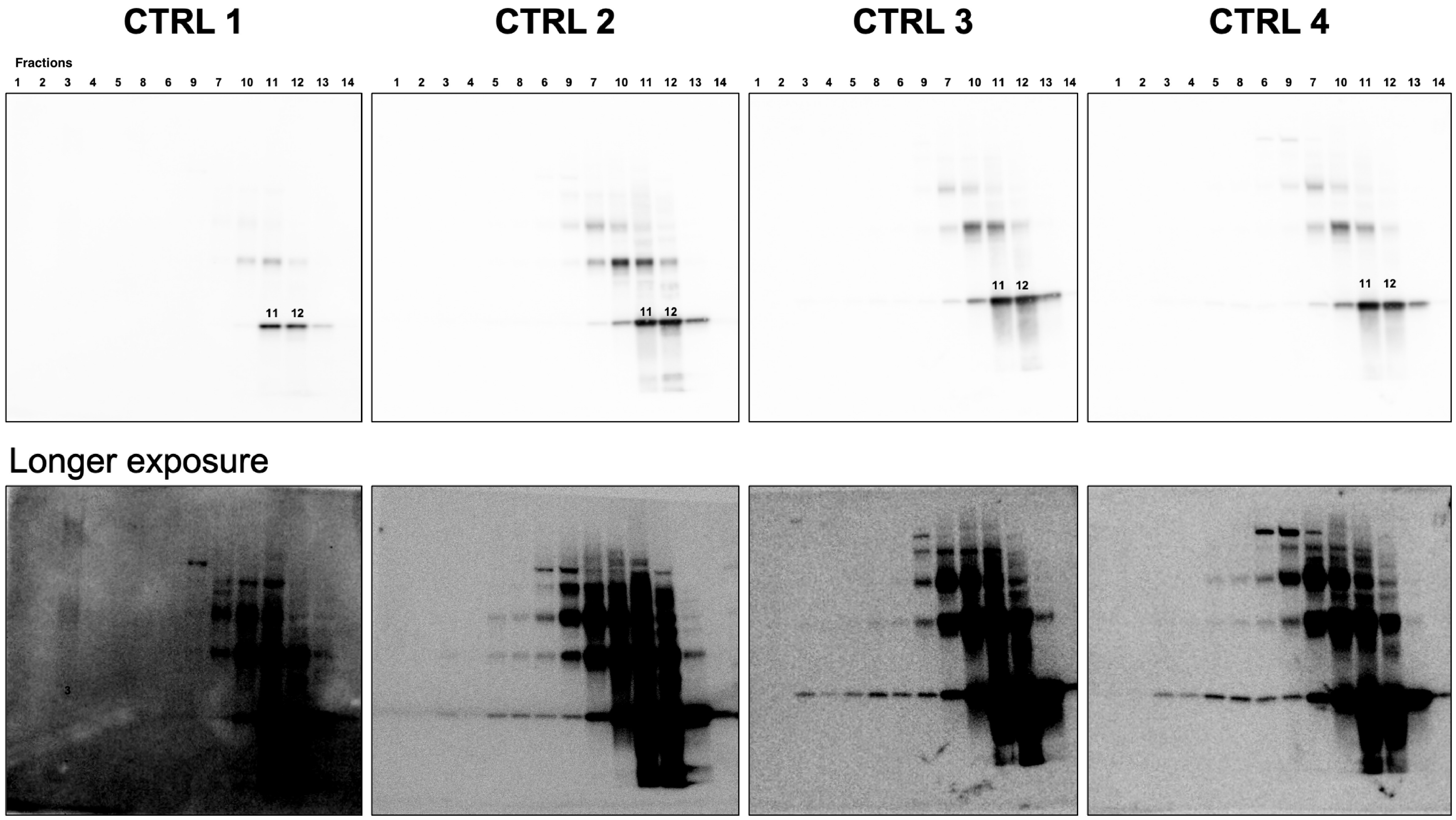

CTRL 1
CTRL 2
CTRL 3
CTRL 4
Fractions
1 2 3 4 5 8 6 9 7 10 11 12 13 14
11 12
Longer exposure

b)

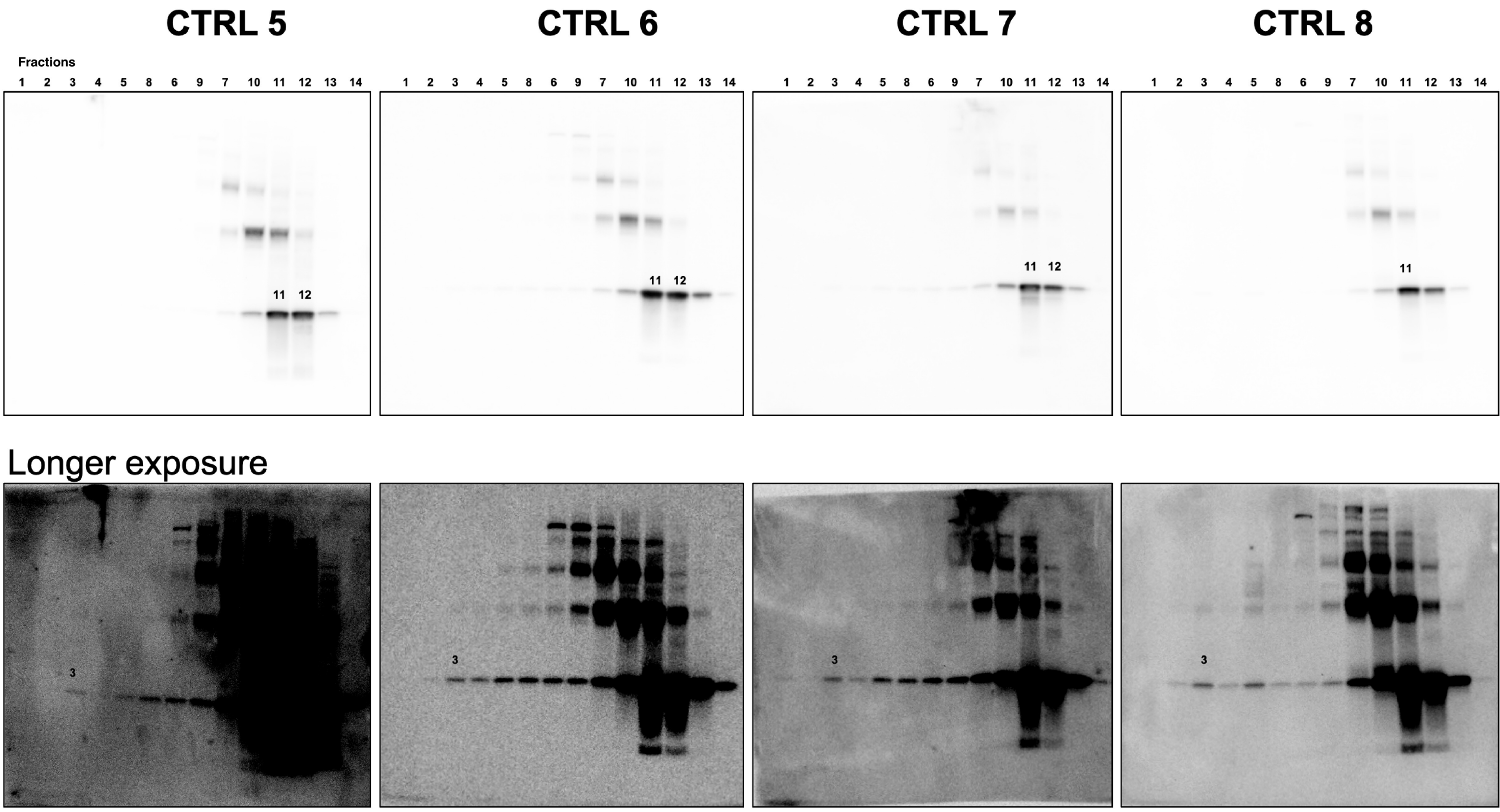

CTRL 5
CTRL 6
CTRL 7
CTRL 8
Fractions
1 2 3 4 5 8 6 9 7 10 11 12 13 14
11 12
Longer exposure

c)

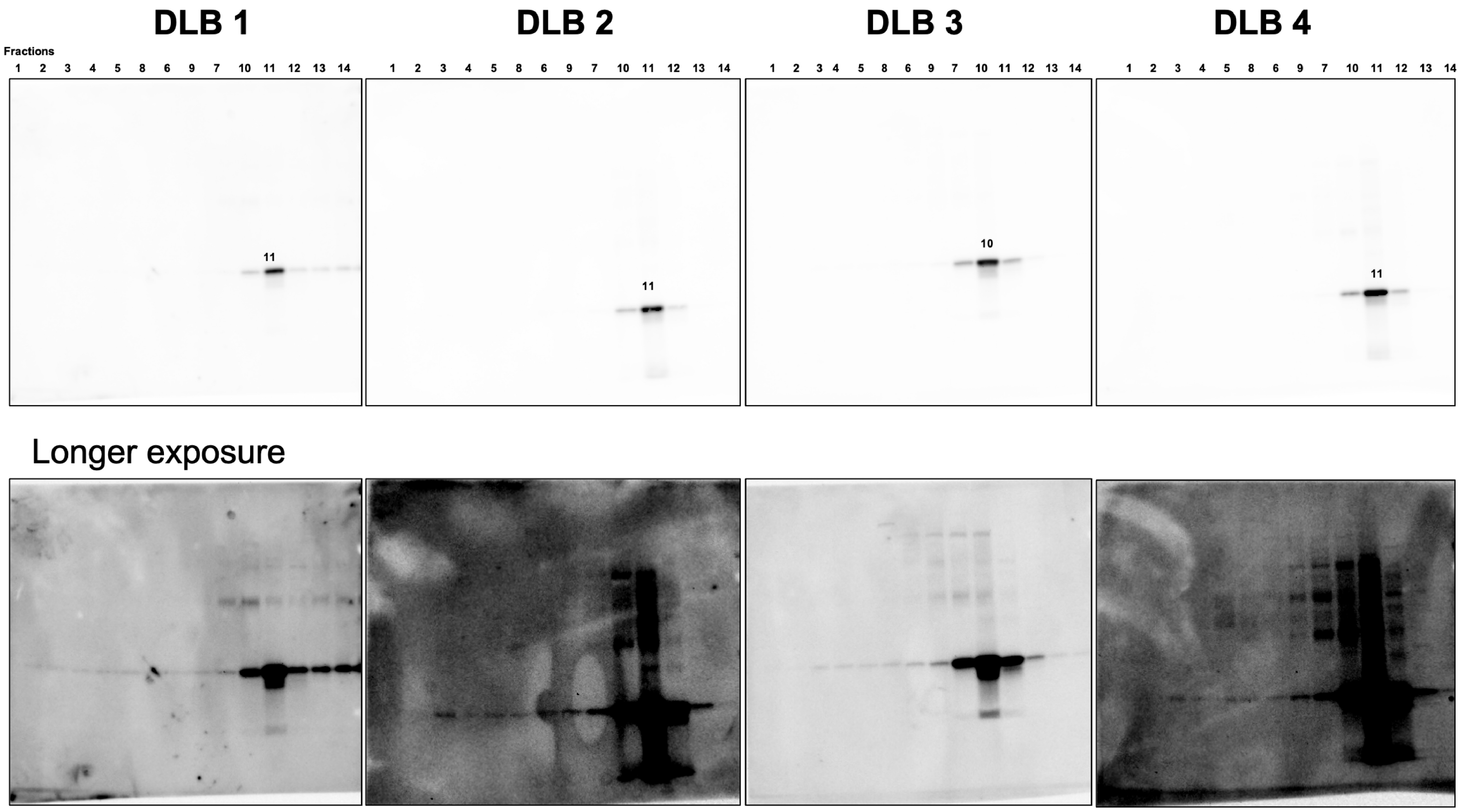
DLB 1
DLB 2
DLB 3
DLB 4
Fractions
1 2 3 4 5 8 6 9 7 10 11 12 13 14
11
11
10
11
Longer exposure

d)

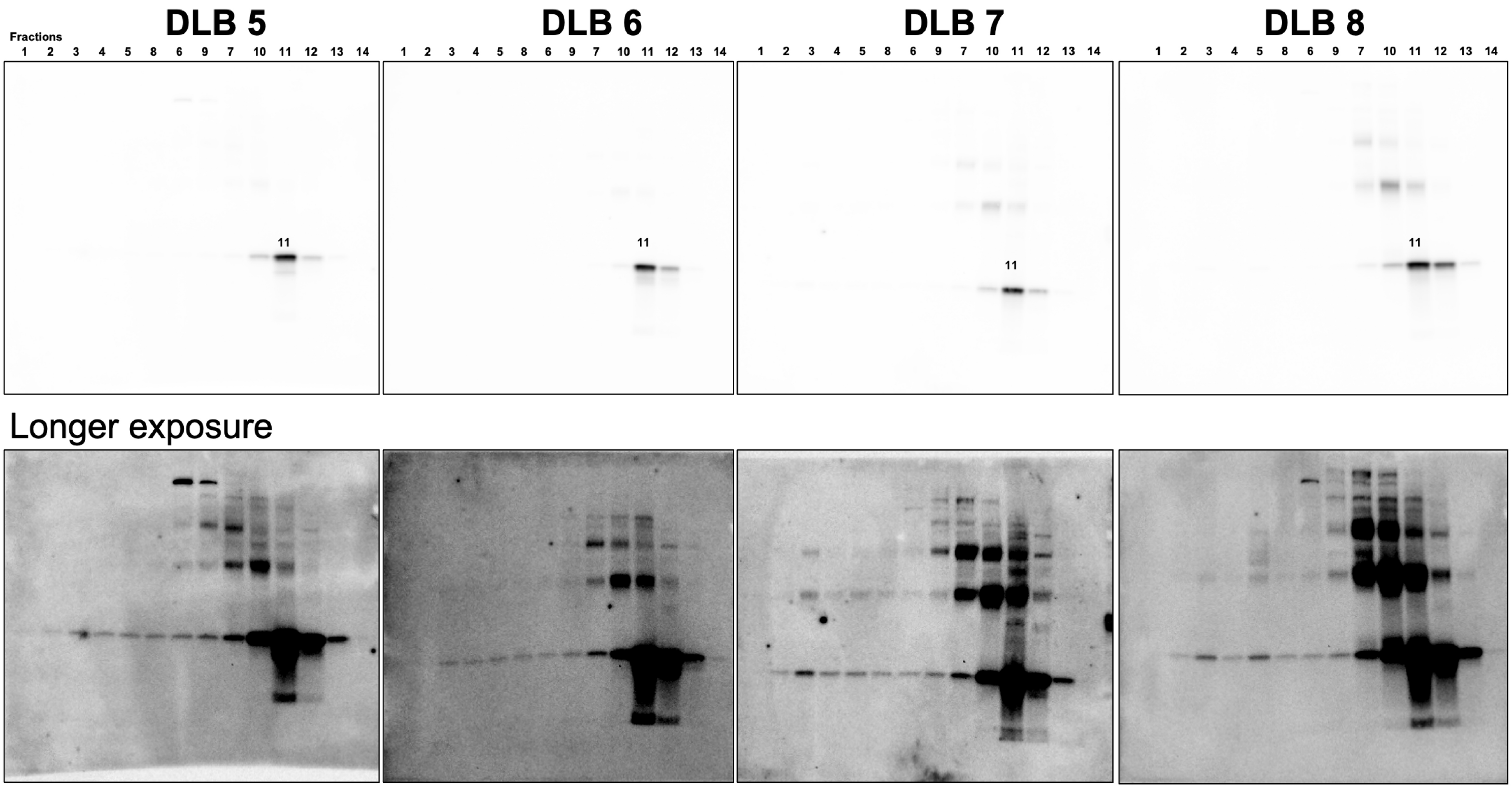
DLB 5
DLB 6
DLB 7
DLB 8
Fractions
1 2 3 4 5 8 6 9 7 10 11 12 13 14
11
11
11
11
Longer exposure

Supplementary figure 1 – Immunoblots from all controls and DLB patients

a-b) Immunoblots from all controls (CTRL #1-8) and c-d) DLB patients (DLB #1-8). Longer exposures of the immunoblots are provided to visualise the dominant SDS-sensitive 17 kDa band in the HMW a-syn pool in some control and DLB patients. .
